# Factors associated with gender-based violence in couples during confinement by COVID-19 in Veracruz

**DOI:** 10.1093/eurpub/ckac131.278

**Published:** 2022-10-25

**Authors:** AJ Cruz Garduza, AR Rodriguez Barranco, R Vazquez Garate, S Uscanga Alcantara, A Martinez Torralba, M Perez Santamaria, H Juarez de la Huerta, JJ Pavan Gallardo, JE Villegas Dominguez

**Affiliations:** 1 Facultad de Medicina, Campus Veracruz, Universidad Veracruzana, Veracruz, Mexico; 2 Facultad de Medicina, Universidad del Valle de Mexico, Veracruz, Mexico

## Abstract

**Background:**

In the world, about a third of women who have had a relationship have suffered some type of violence and Veracruz city in Mexico presented 83 femicides, 519 assaults and 686 disappearances, occupying the 2nd place national in this field.

**Objetives:**

To determinate the factors associated with gender violence during confinement by covid-19

**Methods:**

A cross-sectional, prospective, analytical and observational study was conducted between August - December 2021. Women residents of Veracruz who have or have had a relationship in the last year were included. Violence was quantified by applying to through Google Forms® assessment inventory of mistreatment of women by their couple (APCM) with cronbach’s alpha of 0.94; this instrument takes into account physical and psychological violence. SPSS v22 software was used for data analysis, X2 test with Odds Ratio (OR) and 95% confidence interval (95%CI) and MannWhitney U test.

**Results:**

A total of 740 women participated, with a mean age of 28.1±12.07, 87.8% heterosexual. Gender, sexual preference, educational level, witnessing violence in the family or in relationships with friends obtained values of p > 0.05 to suffer violence, while age was higher for those who suffered gender-based violence (28.8 vs 24.2) and the associated factors (OR/CI95%) were being a housewife (3.1/1.4-6.9), being a student (0.5/0.3-0.8), being married (1.8/1.05-3.3), having a boyfriend (0.3/0.2-0.5), having suffered violence in a previous relationship (1.6/1.06-2.5), identifying gender-based violence correctly or perceived (0.2/0.1-0.4), having suffered gender-based violence at some time by their partner in their last relationship (15.9/5.0-50.9) (p < 0.05)

**Conclusions:**

Being a housewife, being married, having suffered violence in previous relationships and in the last relationship increase the risk of gender violence, while being a student, having a boyfriend and knowing the concept of gender violence decrease the probability of suffering it.

**Key messages:**

• We must work on a deconstruction of ideas that allows women with risk factors for violence with their couples to identify it without fear of not meeting the socio-cultural expectations assigned to it.

• Including a woman’s partner within the structure of prevention of violence against women must be fundamental to promote an environment free of violence.

